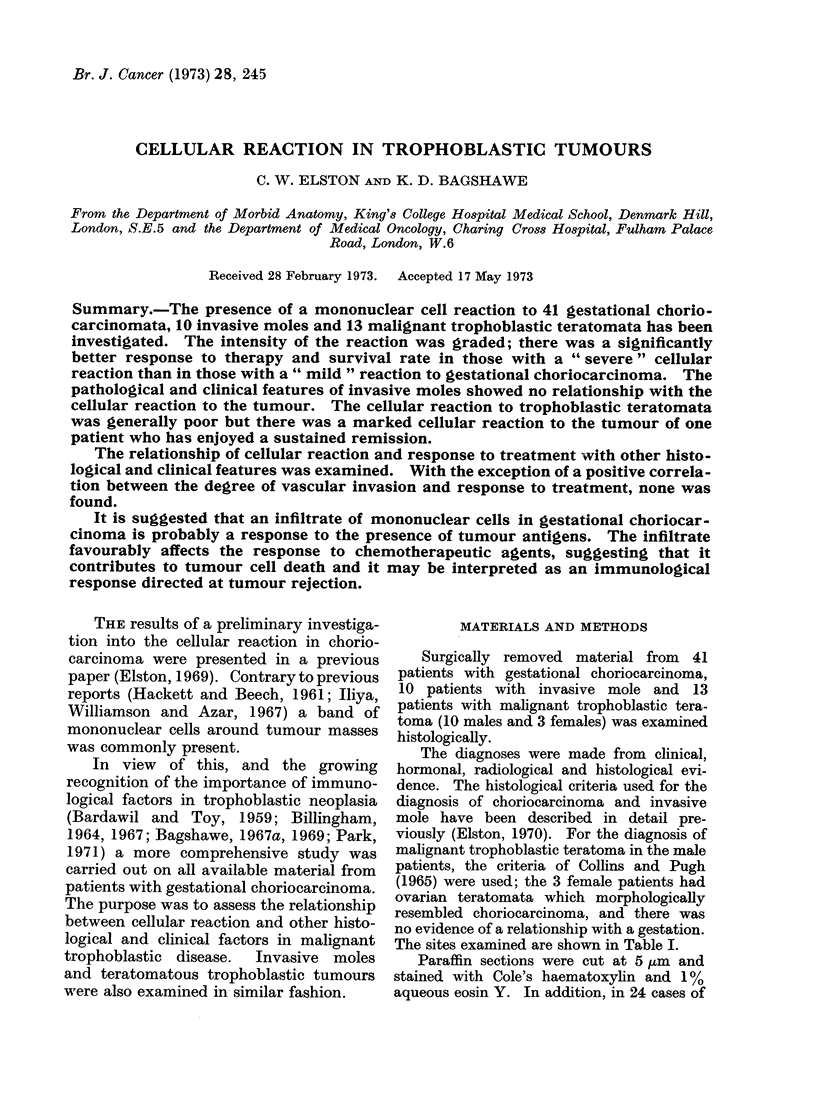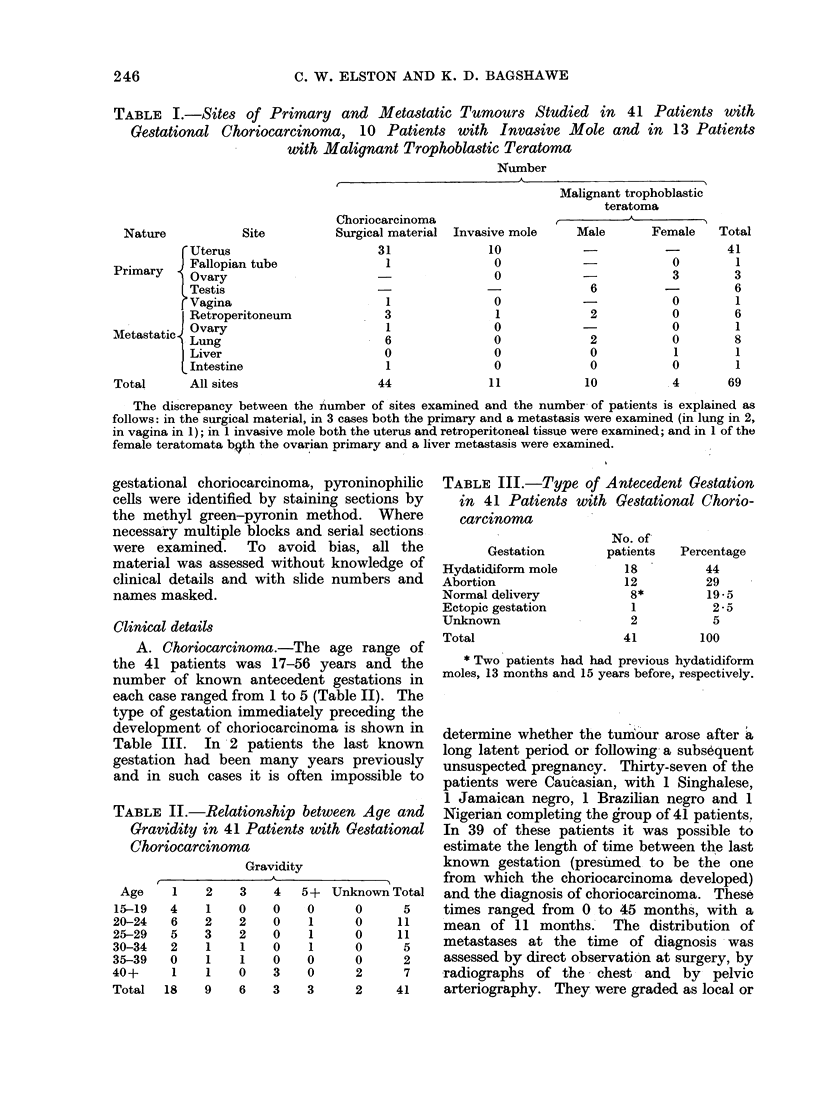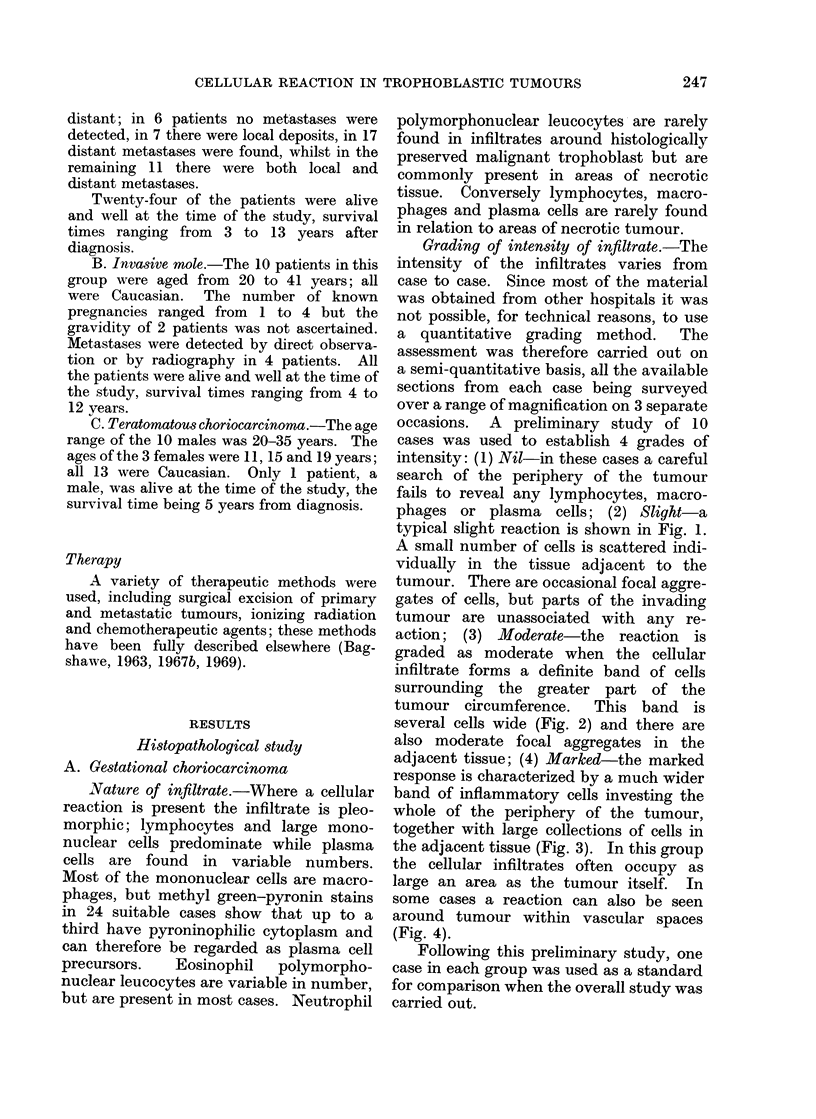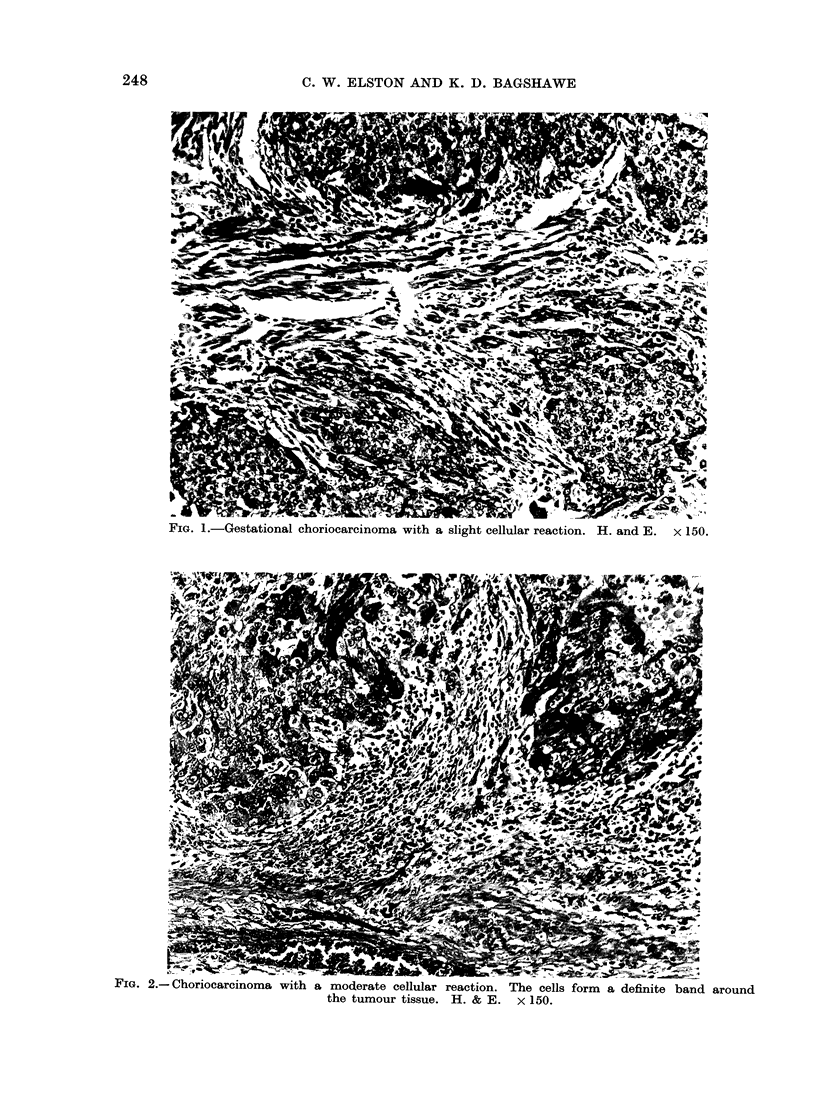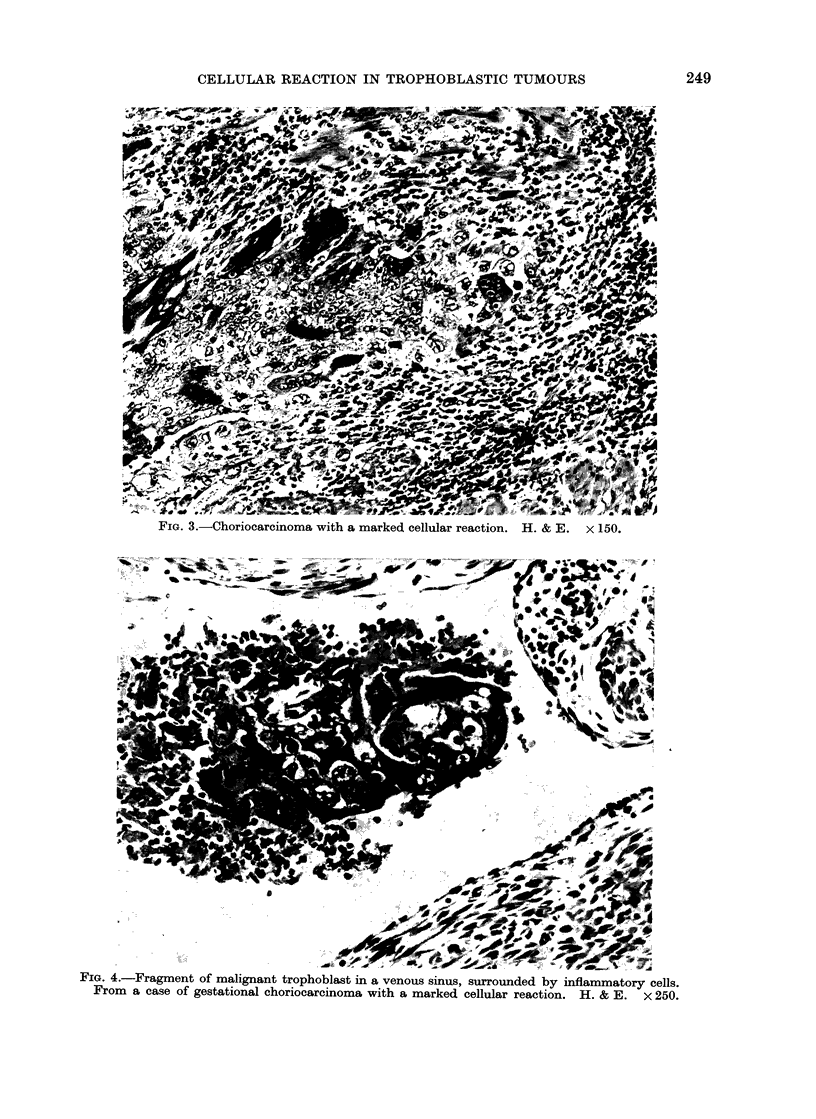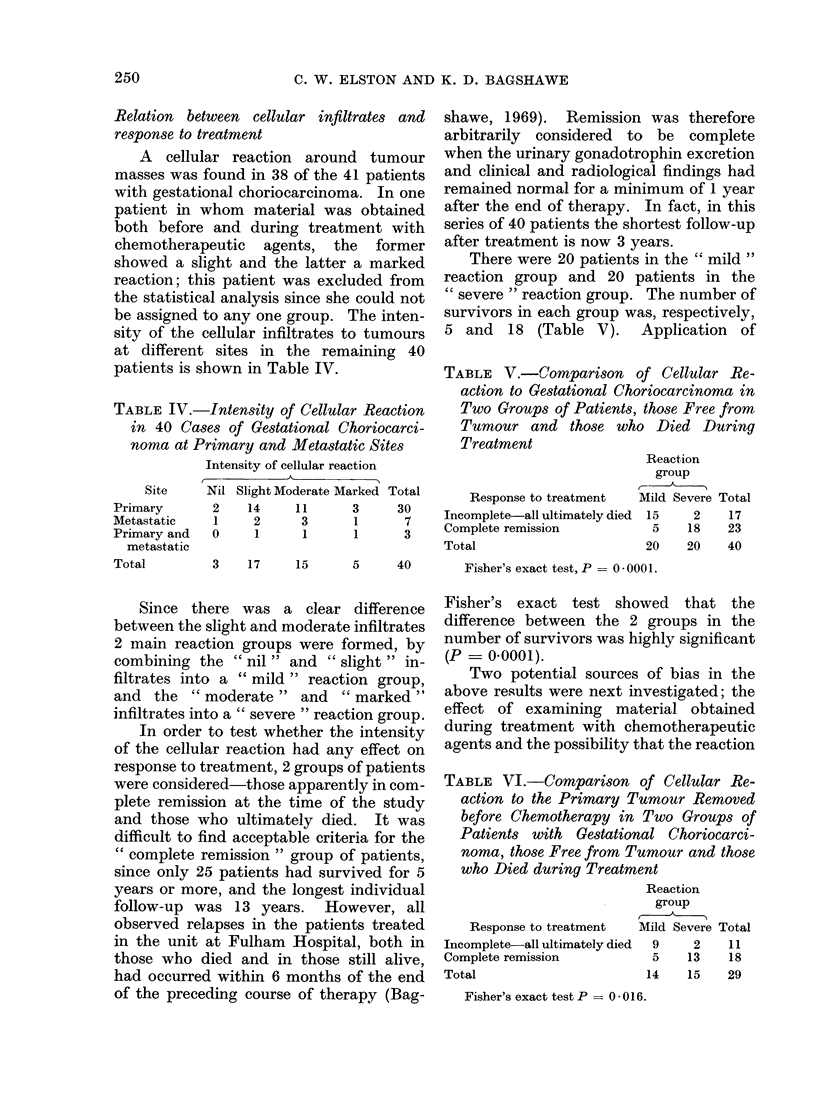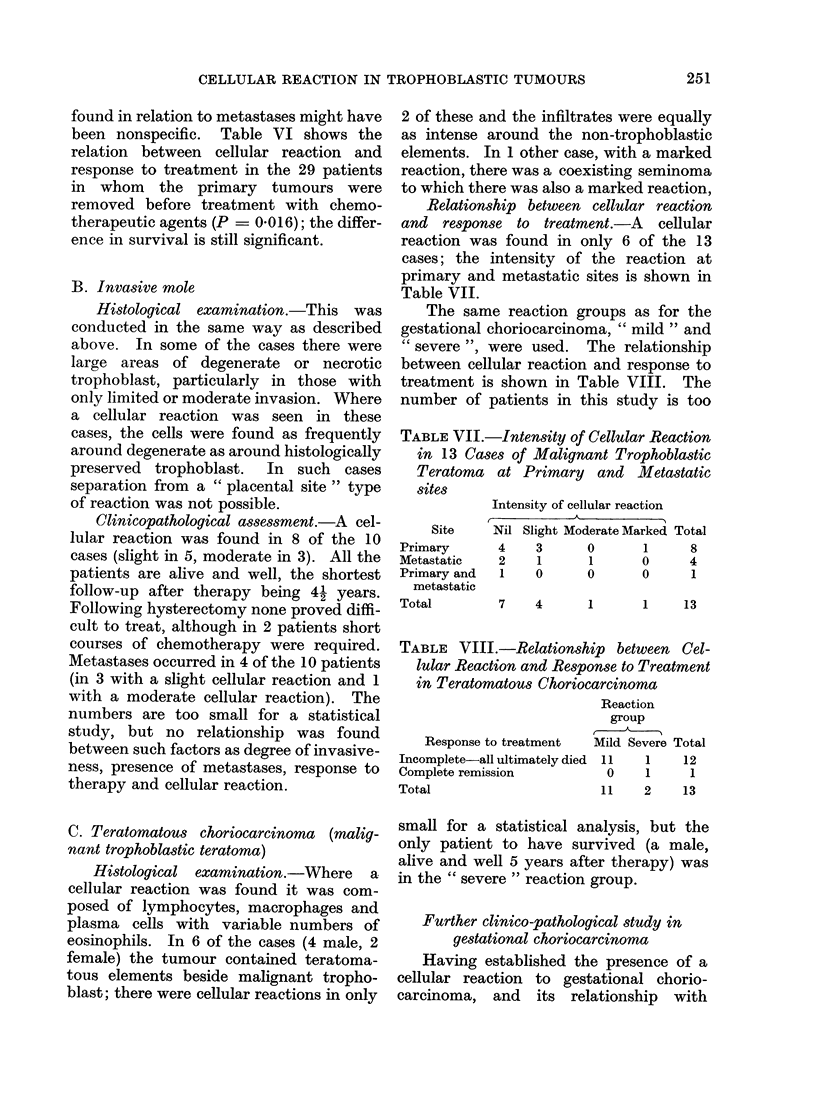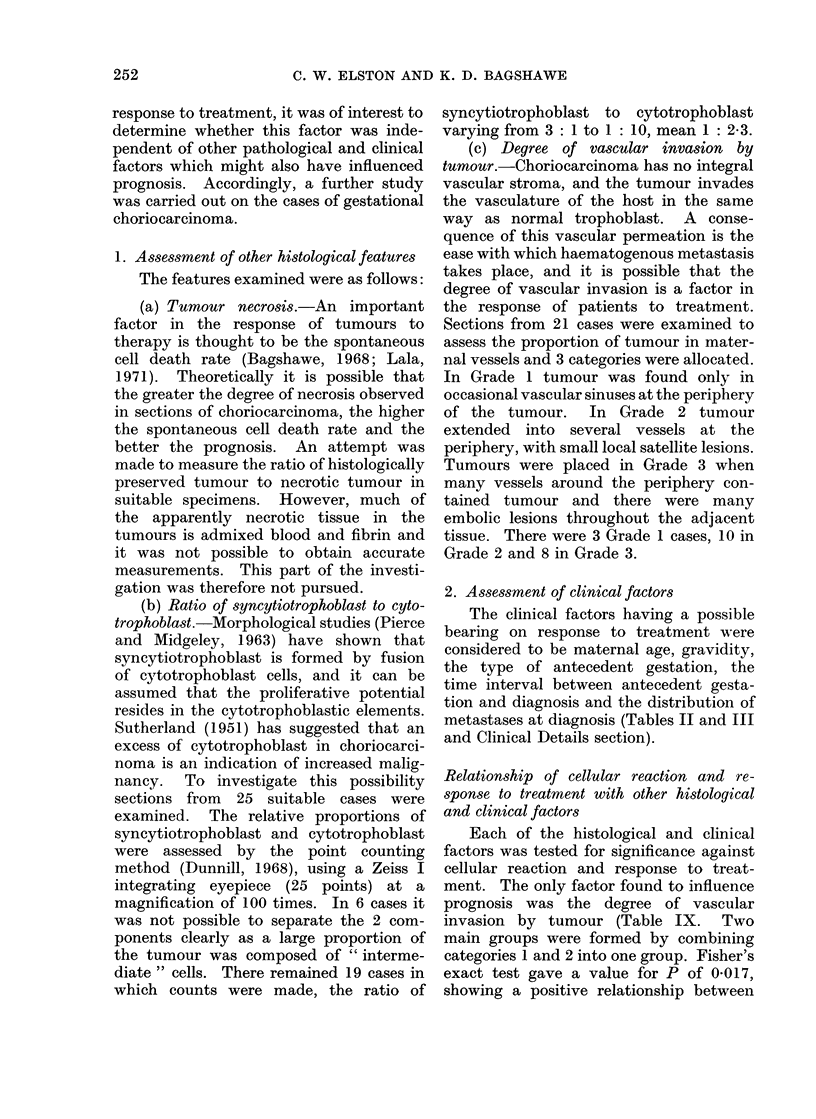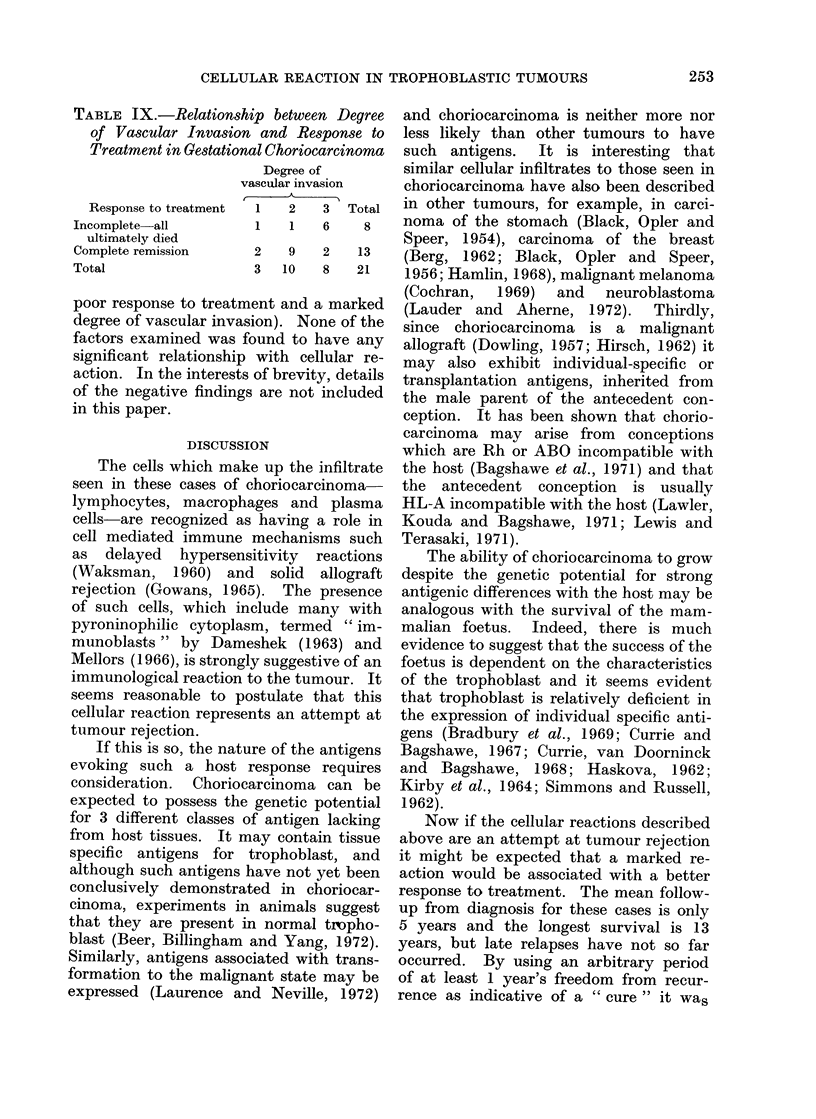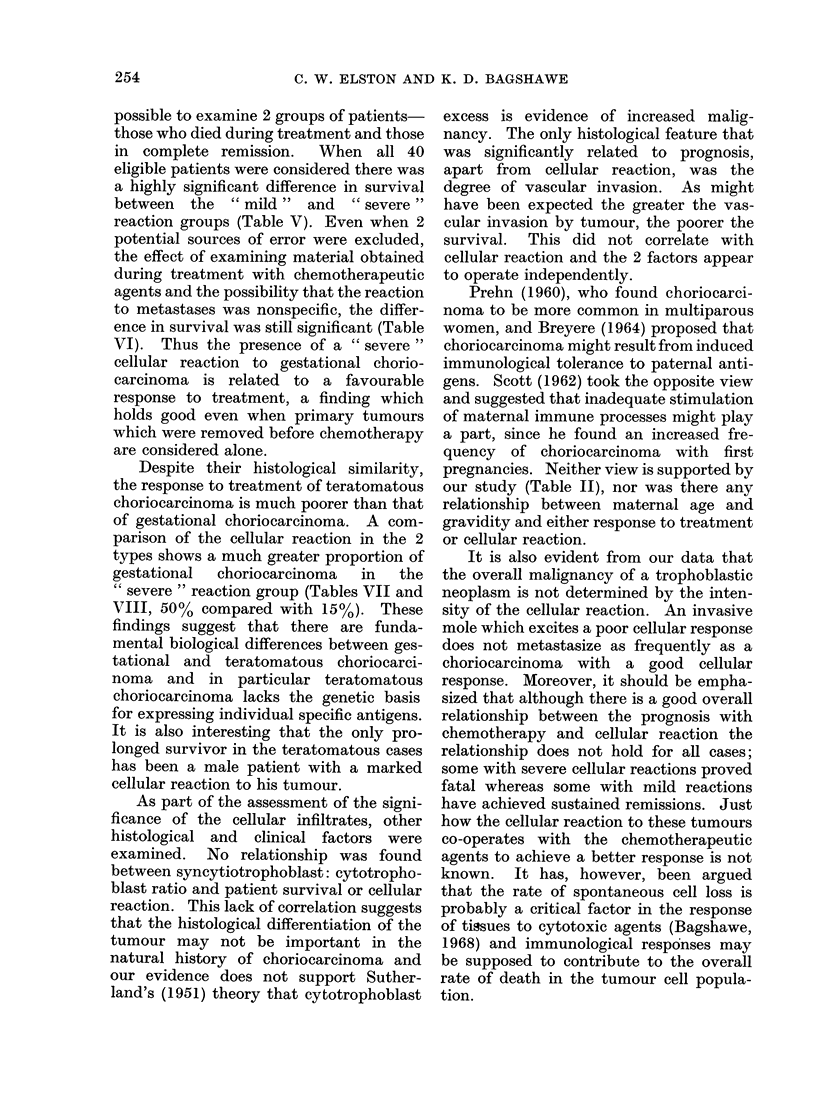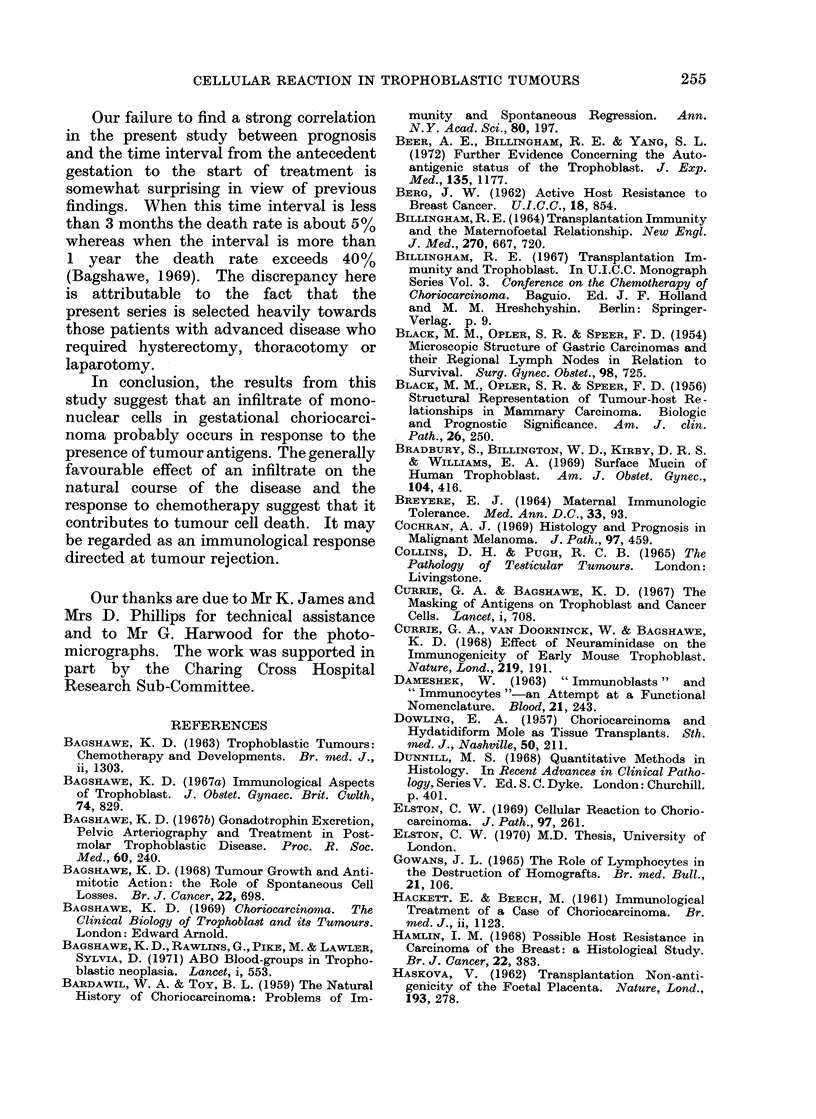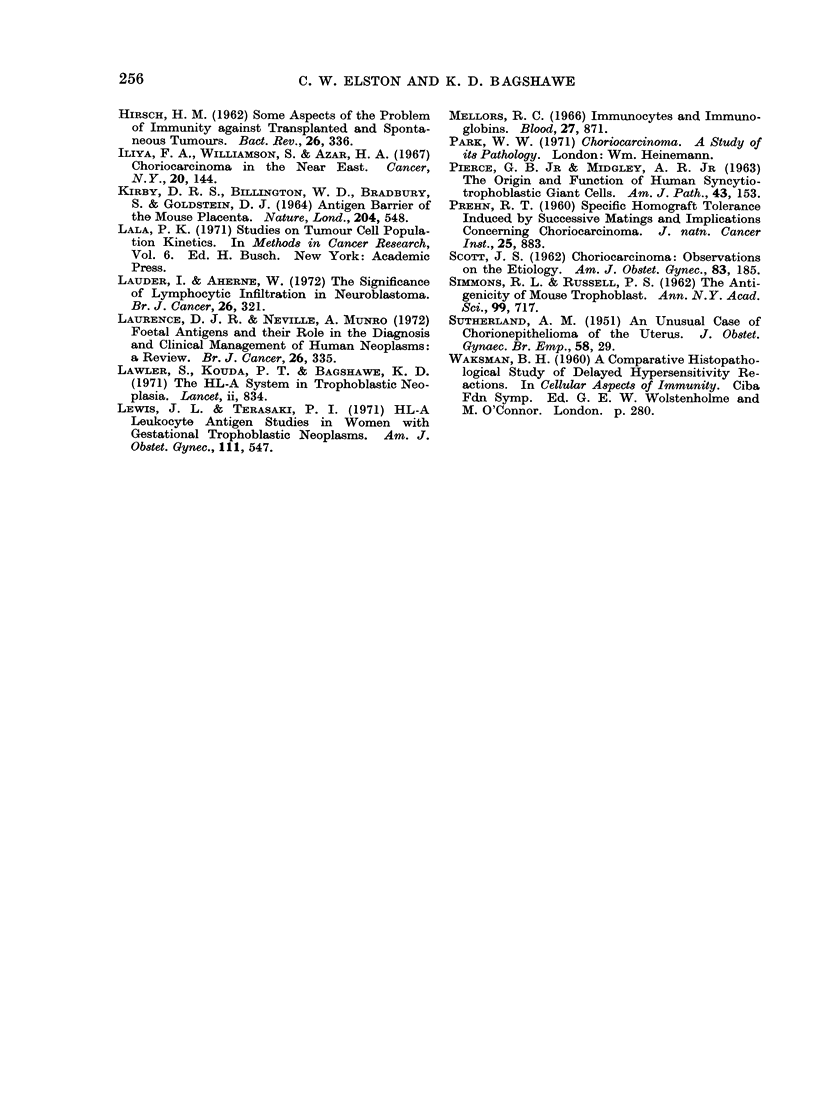# Cellular Reaction in Trophoblastic Tumours

**DOI:** 10.1038/bjc.1973.144

**Published:** 1973-09

**Authors:** C. W. Elston, K. D. Bagshawe

## Abstract

**Images:**


*Br. J. Cancer* (1973) 28, 245

### CELLULAR REACTION IN TROPHOBLASTIC TUMOURS

C. W. ELSTON AND K. D. BAGSHAWE

*From the Department of Morbid Anatomy, King's College Hospital Medical School, Denmark Hill, London, S.E.5 and the Department of Medical Oncology, Charing Cross Hospital, Fulham Palace Road, London, W.6*

Received 28 February 1973. Accepted 17 May 1973

**Summary.—The presence of a mononuclear cell reaction to 41 gestational choriocarcinomata, 10 invasive moles and 13 malignant trophoblastic teratomata has been investigated. The intensity of the reaction was graded; there was a significantly better response to therapy and survival rate in those with a " severe " cellular reaction than in those with a " mild " reaction to gestational choriocarcinoma. The pathological and clinical features of invasive moles showed no relationship with the cellular reaction to the tumour. The cellular reaction to trophoblastic teratomata was generally poor but there was a marked cellular reaction to the tumour of one patient who has enjoyed a sustained remission.**

**The relationship of cellular reaction and response to treatment with other histological and clinical features was examined. With the exception of a positive correlation between the degree of vascular invasion and response to treatment, none was found.**

**It is suggested that an infiltrate of mononuclear cells in gestational choriocarcinoma is probably a response to the presence of tumour antigens. The infiltrate favourably affects the response to chemotherapeutic agents, suggesting that it contributes to tumour cell death and it may be interpreted as an immunological response directed at tumour rejection.**

THE results of a preliminary investigation into the cellular reaction in choriocarcinoma were presented in a previous paper (Elston, 1969). Contrary to previous reports (Hackett and Beech, 1961; Iliya, Williamson and Azar, 1967) a band of mononuclear cells around tumour masses was commonly present.

In view of this, and the growing recognition of the importance of immunological factors in trophoblastic neoplasia (Bardawil and Toy, 1959; Billingham, 1964, 1967; Bagshawe, 1967*a*, 1969; Park, 1971) a more comprehensive study was carried out on all available material from patients with gestational choriocarcinoma. The purpose was to assess the relationship between cellular reaction and other histological and clinical factors in malignant trophoblastic disease. Invasive moles and teratomatous trophoblastic tumours were also examined in similar fashion.

### MATERIALS AND METHODS

Surgically removed material from 41 patients with gestational choriocarcinoma, 10 patients with invasive mole and 13 patients with malignant trophoblastic teratoma (10 males and 3 females) was examined histologically.

The diagnoses were made from clinical, hormonal, radiological and histological evidence. The histological criteria used for the diagnosis of choriocarcinoma and invasive mole have been described in detail previously (Elston, 1970). For the diagnosis of malignant trophoblastic teratoma in the male patients, the criteria of Collins and Pugh (1965) were used; the 3 female patients had ovarian teratomata which morphologically resembled choriocarcinoma, and there was no evidence of a relationship with a gestation. The sites examined are shown in Table I.

Paraffin sections were cut at 5 $\mu$m and stained with Cole's haematoxylin and 1% aqueous eosin Y. In addition, in 24 cases of

TABLE I.—*Sites of Primary and Metastatic Tumours Studied in 41 Patients with Gestational Choriocarcinoma, 10 Patients with Invasive Mole and in 13 Patients with Malignant Trophoblastic Teratoma*

| Nature | Site | Number: Choriocarcinoma Surgical material | Invasive mole | Malignant trophoblastic teratoma: Male | Female | Total |
|---|---|---|---|---|---|---|
| Primary | Uterus | 31 | 10 | — | — | 41 |
| | Fallopian tube | 1 | 0 | — | 0 | 1 |
| | Ovary | — | 0 | — | 3 | 3 |
| | Testis | — | — | 6 | — | 6 |
| Metastatic | Vagina | 1 | 0 | — | 0 | 1 |
| | Retroperitoneum | 3 | 1 | 2 | 0 | 6 |
| | Ovary | 1 | 0 | — | 0 | 1 |
| | Lung | 6 | 0 | 2 | 0 | 8 |
| | Liver | 0 | 0 | 0 | 1 | 1 |
| | Intestine | 1 | 0 | 0 | 0 | 1 |
| Total | All sites | 44 | 11 | 10 | 4 | 69 |

The discrepancy between the number of sites examined and the number of patients is explained as follows: in the surgical material, in 3 cases both the primary and a metastasis were examined (in lung in 2, in vagina in 1); in 1 invasive mole both the uterus and retroperitoneal tissue were examined; and in 1 of the female teratomata both the ovarian primary and a liver metastasis were examined.

gestational choriocarcinoma, pyroninophilic cells were identified by staining sections by the methyl green–pyronin method. Where necessary multiple blocks and serial sections were examined. To avoid bias, all the material was assessed without knowledge of clinical details and with slide numbers and names masked.

*Clinical details*

A. *Choriocarcinoma.*—The age range of the 41 patients was 17–56 years and the number of known antecedent gestations in each case ranged from 1 to 5 (Table II). The type of gestation immediately preceding the development of choriocarcinoma is shown in Table III. In 2 patients the last known gestation had been many years previously and in such cases it is often impossible to determine whether the tumour arose after a long latent period or following a subsequent unsuspected pregnancy. Thirty-seven of the patients were Caucasian, with 1 Singhalese, 1 Jamaican negro, 1 Brazilian negro and 1 Nigerian completing the group of 41 patients. In 39 of these patients it was possible to estimate the length of time between the last known gestation (presumed to be the one from which the choriocarcinoma developed) and the diagnosis of choriocarcinoma. These times ranged from 0 to 45 months, with a mean of 11 months. The distribution of metastases at the time of diagnosis was assessed by direct observation at surgery, by radiographs of the chest and by pelvic arteriography. They were graded as local or

TABLE II.—*Relationship between Age and Gravidity in 41 Patients with Gestational Choriocarcinoma*

| Age | Gravidity: 1 | 2 | 3 | 4 | 5+ | Unknown | Total |
|---|---|---|---|---|---|---|---|
| 15–19 | 4 | 1 | 0 | 0 | 0 | 0 | 5 |
| 20–24 | 6 | 2 | 2 | 0 | 1 | 0 | 11 |
| 25–29 | 5 | 3 | 2 | 0 | 1 | 0 | 11 |
| 30–34 | 2 | 1 | 1 | 0 | 1 | 0 | 5 |
| 35–39 | 0 | 1 | 1 | 0 | 0 | 0 | 2 |
| 40+ | 1 | 1 | 0 | 3 | 0 | 2 | 7 |
| Total | 18 | 9 | 6 | 3 | 3 | 2 | 41 |

TABLE III.—*Type of Antecedent Gestation in 41 Patients with Gestational Choriocarcinoma*

| Gestation | No. of patients | Percentage |
|---|---|---|
| Hydatidiform mole | 18 | 44 |
| Abortion | 12 | 29 |
| Normal delivery | 8* | 19·5 |
| Ectopic gestation | 1 | 2·5 |
| Unknown | 2 | 5 |
| Total | 41 | 100 |

* Two patients had had previous hydatidiform moles, 13 months and 15 years before, respectively.

distant; in 6 patients no metastases were detected, in 7 there were local deposits, in 17 distant metastases were found, whilst in the remaining 11 there were both local and distant metastases.

Twenty-four of the patients were alive and well at the time of the study, survival times ranging from 3 to 13 years after diagnosis.

B. *Invasive mole.*—The 10 patients in this group were aged from 20 to 41 years; all were Caucasian. The number of known pregnancies ranged from 1 to 4 but the gravidity of 2 patients was not ascertained. Metastases were detected by direct observation or by radiography in 4 patients. All the patients were alive and well at the time of the study, survival times ranging from 4 to 12 years.

C. *Teratomatous choriocarcinoma.*—The age range of the 10 males was 20–35 years. The ages of the 3 females were 11, 15 and 19 years; all 13 were Caucasian. Only 1 patient, a male, was alive at the time of the study, the survival time being 5 years from diagnosis.

### *Therapy*

A variety of therapeutic methods were used, including surgical excision of primary and metastatic tumours, ionizing radiation and chemotherapeutic agents; these methods have been fully described elsewhere (Bagshawe, 1963, 1967*b*, 1969).

### RESULTS

### *Histopathological study*

#### A. *Gestational choriocarcinoma*

*Nature of infiltrate.*—Where a cellular reaction is present the infiltrate is pleomorphic; lymphocytes and large mononuclear cells predominate while plasma cells are found in variable numbers. Most of the mononuclear cells are macrophages, but methyl green–pyronin stains in 24 suitable cases show that up to a third have pyroninophilic cytoplasm and can therefore be regarded as plasma cell precursors. Eosinophil polymorphonuclear leucocytes are variable in number, but are present in most cases. Neutrophil polymorphonuclear leucocytes are rarely found in infiltrates around histologically preserved malignant trophoblast but are commonly present in areas of necrotic tissue. Conversely lymphocytes, macrophages and plasma cells are rarely found in relation to areas of necrotic tumour.

*Grading of intensity of infiltrate.*—The intensity of the infiltrates varies from case to case. Since most of the material was obtained from other hospitals it was not possible, for technical reasons, to use a quantitative grading method. The assessment was therefore carried out on a semi-quantitative basis, all the available sections from each case being surveyed over a range of magnification on 3 separate occasions. A preliminary study of 10 cases was used to establish 4 grades of intensity: (1) *Nil*—in these cases a careful search of the periphery of the tumour fails to reveal any lymphocytes, macrophages or plasma cells; (2) *Slight*—a typical slight reaction is shown in Fig. 1. A small number of cells is scattered individually in the tissue adjacent to the tumour. There are occasional focal aggregates of cells, but parts of the invading tumour are unassociated with any reaction; (3) *Moderate*—the reaction is graded as moderate when the cellular infiltrate forms a definite band of cells surrounding the greater part of the tumour circumference. This band is several cells wide (Fig. 2) and there are also moderate focal aggregates in the adjacent tissue; (4) *Marked*—the marked response is characterized by a much wider band of inflammatory cells investing the whole of the periphery of the tumour, together with large collections of cells in the adjacent tissue (Fig. 3). In this group the cellular infiltrates often occupy as large an area as the tumour itself. In some cases a reaction can also be seen around tumour within vascular spaces (Fig. 4).

Following this preliminary study, one case in each group was used as a standard for comparison when the overall study was carried out.

FIG. 1.—Gestational choriocarcinoma with a slight cellular reaction. H. and E. ×150.

FIG. 2.—Choriocarcinoma with a moderate cellular reaction. The cells form a definite band around the tumour tissue. H. & E. ×150.

FIG. 3.—Choriocarcinoma with a marked cellular reaction. H. & E. ×150.

FIG. 4.—Fragment of malignant trophoblast in a venous sinus, surrounded by inflammatory cells. From a case of gestational choriocarcinoma with a marked cellular reaction. H. & E. ×250.

*Relation between cellular infiltrates and response to treatment*

A cellular reaction around tumour masses was found in 38 of the 41 patients with gestational choriocarcinoma. In one patient in whom material was obtained both before and during treatment with chemotherapeutic agents, the former showed a slight and the latter a marked reaction; this patient was excluded from the statistical analysis since she could not be assigned to any one group. The intensity of the cellular infiltrates to tumours at different sites in the remaining 40 patients is shown in Table IV.

TABLE IV.—*Intensity of Cellular Reaction in 40 Cases of Gestational Choriocarcinoma at Primary and Metastatic Sites*

| | Intensity of cellular reaction | | | | |
|---|---|---|---|---|---|
| Site | Nil | Slight | Moderate | Marked | Total |
| Primary | 2 | 14 | 11 | 3 | 30 |
| Metastatic | 1 | 2 | 3 | 1 | 7 |
| Primary and metastatic | 0 | 1 | 1 | 1 | 3 |
| Total | 3 | 17 | 15 | 5 | 40 |

Since there was a clear difference between the slight and moderate infiltrates 2 main reaction groups were formed, by combining the " nil " and " slight " infiltrates into a " mild " reaction group, and the " moderate " and " marked " infiltrates into a " severe " reaction group.

In order to test whether the intensity of the cellular reaction had any effect on response to treatment, 2 groups of patients were considered—those apparently in complete remission at the time of the study and those who ultimately died. It was difficult to find acceptable criteria for the " complete remission " group of patients, since only 25 patients had survived for 5 years or more, and the longest individual follow-up was 13 years. However, all observed relapses in the patients treated in the unit at Fulham Hospital, both in those who died and in those still alive, had occurred within 6 months of the end of the preceding course of therapy (Bagshawe, 1969). Remission was therefore arbitrarily considered to be complete when the urinary gonadotrophin excretion and clinical and radiological findings had remained normal for a minimum of 1 year after the end of therapy. In fact, in this series of 40 patients the shortest follow-up after treatment is now 3 years.

There were 20 patients in the " mild " reaction group and 20 patients in the " severe " reaction group. The number of survivors in each group was, respectively, 5 and 18 (Table V). Application of Fisher's exact test showed that the difference between the 2 groups in the number of survivors was highly significant ($P = 0 \cdot 0001$).

TABLE V.—*Comparison of Cellular Reaction to Gestational Choriocarcinoma in Two Groups of Patients, those Free from Tumour and those who Died During Treatment*

| | Reaction group | | |
|---|---|---|---|
| Response to treatment | Mild | Severe | Total |
| Incomplete—all ultimately died | 15 | 2 | 17 |
| Complete remission | 5 | 18 | 23 |
| Total | 20 | 20 | 40 |

Fisher's exact test, $P = 0 \cdot 0001$.

Two potential sources of bias in the above results were next investigated; the effect of examining material obtained during treatment with chemotherapeutic agents and the possibility that the reaction

TABLE VI.—*Comparison of Cellular Reaction to the Primary Tumour Removed before Chemotherapy in Two Groups of Patients with Gestational Choriocarcinoma, those Free from Tumour and those who Died during Treatment*

| | Reaction group | | |
|---|---|---|---|
| Response to treatment | Mild | Severe | Total |
| Incomplete—all ultimately died | 9 | 2 | 11 |
| Complete remission | 5 | 13 | 18 |
| Total | 14 | 15 | 29 |

Fisher's exact test $P = 0 \cdot 016$.

found in relation to metastases might have been nonspecific. Table VI shows the relation between cellular reaction and response to treatment in the 29 patients in whom the primary tumours were removed before treatment with chemotherapeutic agents ($P = 0{\cdot}016$); the difference in survival is still significant.

B. *Invasive mole*

*Histological examination.*—This was conducted in the same way as described above. In some of the cases there were large areas of degenerate or necrotic trophoblast, particularly in those with only limited or moderate invasion. Where a cellular reaction was seen in these cases, the cells were found as frequently around degenerate as around histologically preserved trophoblast. In such cases separation from a " placental site " type of reaction was not possible.

*Clinicopathological assessment.*—A cellular reaction was found in 8 of the 10 cases (slight in 5, moderate in 3). All the patients are alive and well, the shortest follow-up after therapy being 4½ years. Following hysterectomy none proved difficult to treat, although in 2 patients short courses of chemotherapy were required. Metastases occurred in 4 of the 10 patients (in 3 with a slight cellular reaction and 1 with a moderate cellular reaction). The numbers are too small for a statistical study, but no relationship was found between such factors as degree of invasiveness, presence of metastases, response to therapy and cellular reaction.

C. *Teratomatous choriocarcinoma* (*malignant trophoblastic teratoma*)

*Histological examination.*—Where a cellular reaction was found it was composed of lymphocytes, macrophages and plasma cells with variable numbers of eosinophils. In 6 of the cases (4 male, 2 female) the tumour contained teratomatous elements beside malignant trophoblast; there were cellular reactions in only 2 of these and the infiltrates were equally as intense around the non-trophoblastic elements. In 1 other case, with a marked reaction, there was a coexisting seminoma to which there was also a marked reaction,

*Relationship between cellular reaction and response to treatment.*—A cellular reaction was found in only 6 of the 13 cases; the intensity of the reaction at primary and metastatic sites is shown in Table VII.

The same reaction groups as for the gestational choriocarcinoma, " mild " and " severe ", were used. The relationship between cellular reaction and response to treatment is shown in Table VIII. The number of patients in this study is too small for a statistical analysis, but the only patient to have survived (a male, alive and well 5 years after therapy) was in the " severe " reaction group.

TABLE VII.—*Intensity of Cellular Reaction in 13 Cases of Malignant Trophoblastic Teratoma at Primary and Metastatic sites*

| Site | Intensity of cellular reaction | | | | |
|---|---|---|---|---|---|
| | Nil | Slight | Moderate | Marked | Total |
| Primary | 4 | 3 | 0 | 1 | 8 |
| Metastatic | 2 | 1 | 1 | 0 | 4 |
| Primary and metastatic | 1 | 0 | 0 | 0 | 1 |
| Total | 7 | 4 | 1 | 1 | 13 |

TABLE VIII.—*Relationship between Cellular Reaction and Response to Treatment in Teratomatous Choriocarcinoma*

| Response to treatment | Reaction group | | |
|---|---|---|---|
| | Mild | Severe | Total |
| Incomplete—all ultimately died | 11 | 1 | 12 |
| Complete remission | 0 | 1 | 1 |
| Total | 11 | 2 | 13 |

### *Further clinico-pathological study in gestational choriocarcinoma*

Having established the presence of a cellular reaction to gestational choriocarcinoma, and its relationship with response to treatment, it was of interest to determine whether this factor was independent of other pathological and clinical factors which might also have influenced prognosis. Accordingly, a further study was carried out on the cases of gestational choriocarcinoma.

1. *Assessment of other histological features*

The features examined were as follows:

(a) *Tumour necrosis.*—An important factor in the response of tumours to therapy is thought to be the spontaneous cell death rate (Bagshawe, 1968; Lala, 1971). Theoretically it is possible that the greater the degree of necrosis observed in sections of choriocarcinoma, the higher the spontaneous cell death rate and the better the prognosis. An attempt was made to measure the ratio of histologically preserved tumour to necrotic tumour in suitable specimens. However, much of the apparently necrotic tissue in the tumours is admixed blood and fibrin and it was not possible to obtain accurate measurements. This part of the investigation was therefore not pursued.

(b) *Ratio of syncytiotrophoblast to cytotrophoblast.*—Morphological studies (Pierce and Midgeley, 1963) have shown that syncytiotrophoblast is formed by fusion of cytotrophoblast cells, and it can be assumed that the proliferative potential resides in the cytotrophoblastic elements. Sutherland (1951) has suggested that an excess of cytotrophoblast in choriocarcinoma is an indication of increased malignancy. To investigate this possibility sections from 25 suitable cases were examined. The relative proportions of syncytiotrophoblast and cytotrophoblast were assessed by the point counting method (Dunnill, 1968), using a Zeiss I integrating eyepiece (25 points) at a magnification of 100 times. In 6 cases it was not possible to separate the 2 components clearly as a large proportion of the tumour was composed of "intermediate" cells. There remained 19 cases in which counts were made, the ratio of syncytiotrophoblast to cytotrophoblast varying from 3 : 1 to 1 : 10, mean 1 : 2·3.

(c) *Degree of vascular invasion by tumour.*—Choriocarcinoma has no integral vascular stroma, and the tumour invades the vasculature of the host in the same way as normal trophoblast. A consequence of this vascular permeation is the ease with which haematogenous metastasis takes place, and it is possible that the degree of vascular invasion is a factor in the response of patients to treatment. Sections from 21 cases were examined to assess the proportion of tumour in maternal vessels and 3 categories were allocated. In Grade 1 tumour was found only in occasional vascular sinuses at the periphery of the tumour. In Grade 2 tumour extended into several vessels at the periphery, with small local satellite lesions. Tumours were placed in Grade 3 when many vessels around the periphery contained tumour and there were many embolic lesions throughout the adjacent tissue. There were 3 Grade 1 cases, 10 in Grade 2 and 8 in Grade 3.

2. *Assessment of clinical factors*

The clinical factors having a possible bearing on response to treatment were considered to be maternal age, gravidity, the type of antecedent gestation, the time interval between antecedent gestation and diagnosis and the distribution of metastases at diagnosis (Tables II and III and Clinical Details section).

*Relationship of cellular reaction and response to treatment with other histological and clinical factors*

Each of the histological and clinical factors was tested for significance against cellular reaction and response to treatment. The only factor found to influence prognosis was the degree of vascular invasion by tumour (Table IX. Two main groups were formed by combining categories 1 and 2 into one group. Fisher's exact test gave a value for $P$ of 0·017, showing a positive relationship between

TABLE IX.—*Relationship between Degree of Vascular Invasion and Response to Treatment in Gestational Choriocarcinoma*

| Response to treatment | Degree of vascular invasion 1 | 2 | 3 | Total |
|---|---|---|---|---|
| Incomplete—all ultimately died | 1 | 1 | 6 | 8 |
| Complete remission | 2 | 9 | 2 | 13 |
| Total | 3 | 10 | 8 | 21 |

poor response to treatment and a marked degree of vascular invasion). None of the factors examined was found to have any significant relationship with cellular reaction. In the interests of brevity, details of the negative findings are not included in this paper.

### DISCUSSION

The cells which make up the infiltrate seen in these cases of choriocarcinoma—lymphocytes, macrophages and plasma cells—are recognized as having a role in cell mediated immune mechanisms such as delayed hypersensitivity reactions (Waksman, 1960) and solid allograft rejection (Gowans, 1965). The presence of such cells, which include many with pyroninophilic cytoplasm, termed " immunoblasts " by Dameshek (1963) and Mellors (1966), is strongly suggestive of an immunological reaction to the tumour. It seems reasonable to postulate that this cellular reaction represents an attempt at tumour rejection.

If this is so, the nature of the antigens evoking such a host response requires consideration. Choriocarcinoma can be expected to possess the genetic potential for 3 different classes of antigen lacking from host tissues. It may contain tissue specific antigens for trophoblast, and although such antigens have not yet been conclusively demonstrated in choriocarcinoma, experiments in animals suggest that they are present in normal trophoblast (Beer, Billingham and Yang, 1972). Similarly, antigens associated with transformation to the malignant state may be expressed (Laurence and Neville, 1972) and choriocarcinoma is neither more nor less likely than other tumours to have such antigens. It is interesting that similar cellular infiltrates to those seen in choriocarcinoma have also been described in other tumours, for example, in carcinoma of the stomach (Black, Opler and Speer, 1954), carcinoma of the breast (Berg, 1962; Black, Opler and Speer, 1956; Hamlin, 1968), malignant melanoma (Cochran, 1969) and neuroblastoma (Lauder and Aherne, 1972). Thirdly, since choriocarcinoma is a malignant allograft (Dowling, 1957; Hirsch, 1962) it may also exhibit individual-specific or transplantation antigens, inherited from the male parent of the antecedent conception. It has been shown that choriocarcinoma may arise from conceptions which are Rh or ABO incompatible with the host (Bagshawe *et al.*, 1971) and that the antecedent conception is usually HL-A incompatible with the host (Lawler, Kouda and Bagshawe, 1971; Lewis and Terasaki, 1971).

The ability of choriocarcinoma to grow despite the genetic potential for strong antigenic differences with the host may be analogous with the survival of the mammalian foetus. Indeed, there is much evidence to suggest that the success of the foetus is dependent on the characteristics of the trophoblast and it seems evident that trophoblast is relatively deficient in the expression of individual specific antigens (Bradbury *et al.*, 1969; Currie and Bagshawe, 1967; Currie, van Doorninck and Bagshawe, 1968; Haskova, 1962; Kirby *et al.*, 1964; Simmons and Russell, 1962).

Now if the cellular reactions described above are an attempt at tumour rejection it might be expected that a marked reaction would be associated with a better response to treatment. The mean follow-up from diagnosis for these cases is only 5 years and the longest survival is 13 years, but late relapses have not so far occurred. By using an arbitrary period of at least 1 year's freedom from recurrence as indicative of a " cure " it was possible to examine 2 groups of patients—those who died during treatment and those in complete remission. When all 40 eligible patients were considered there was a highly significant difference in survival between the " mild " and " severe " reaction groups (Table V). Even when 2 potential sources of error were excluded, the effect of examining material obtained during treatment with chemotherapeutic agents and the possibility that the reaction to metastases was nonspecific, the difference in survival was still significant (Table VI). Thus the presence of a " severe " cellular reaction to gestational choriocarcinoma is related to a favourable response to treatment, a finding which holds good even when primary tumours which were removed before chemotherapy are considered alone.

Despite their histological similarity, the response to treatment of teratomatous choriocarcinoma is much poorer than that of gestational choriocarcinoma. A comparison of the cellular reaction in the 2 types shows a much greater proportion of gestational choriocarcinoma in the " severe " reaction group (Tables VII and VIII, 50% compared with 15%). These findings suggest that there are fundamental biological differences between gestational and teratomatous choriocarcinoma and in particular teratomatous choriocarcinoma lacks the genetic basis for expressing individual specific antigens. It is also interesting that the only prolonged survivor in the teratomatous cases has been a male patient with a marked cellular reaction to his tumour.

As part of the assessment of the significance of the cellular infiltrates, other histological and clinical factors were examined. No relationship was found between syncytiotrophoblast: cytotrophoblast ratio and patient survival or cellular reaction. This lack of correlation suggests that the histological differentiation of the tumour may not be important in the natural history of choriocarcinoma and our evidence does not support Sutherland's (1951) theory that cytotrophoblast excess is evidence of increased malignancy. The only histological feature that was significantly related to prognosis, apart from cellular reaction, was the degree of vascular invasion. As might have been expected the greater the vascular invasion by tumour, the poorer the survival. This did not correlate with cellular reaction and the 2 factors appear to operate independently.

Prehn (1960), who found choriocarcinoma to be more common in multiparous women, and Breyere (1964) proposed that choriocarcinoma might result from induced immunological tolerance to paternal antigens. Scott (1962) took the opposite view and suggested that inadequate stimulation of maternal immune processes might play a part, since he found an increased frequency of choriocarcinoma with first pregnancies. Neither view is supported by our study (Table II), nor was there any relationship between maternal age and gravidity and either response to treatment or cellular reaction.

It is also evident from our data that the overall malignancy of a trophoblastic neoplasm is not determined by the intensity of the cellular reaction. An invasive mole which excites a poor cellular response does not metastasize as frequently as a choriocarcinoma with a good cellular response. Moreover, it should be emphasized that although there is a good overall relationship between the prognosis with chemotherapy and cellular reaction the relationship does not hold for all cases; some with severe cellular reactions proved fatal whereas some with mild reactions have achieved sustained remissions. Just how the cellular reaction to these tumours co-operates with the chemotherapeutic agents to achieve a better response is not known. It has, however, been argued that the rate of spontaneous cell loss is probably a critical factor in the response of tissues to cytotoxic agents (Bagshawe, 1968) and immunological responses may be supposed to contribute to the overall rate of death in the tumour cell population.

Our failure to find a strong correlation in the present study between prognosis and the time interval from the antecedent gestation to the start of treatment is somewhat surprising in view of previous findings. When this time interval is less than 3 months the death rate is about 5% whereas when the interval is more than 1 year the death rate exceeds 40% (Bagshawe, 1969). The discrepancy here is attributable to the fact that the present series is selected heavily towards those patients with advanced disease who required hysterectomy, thoracotomy or laparotomy.

In conclusion, the results from this study suggest that an infiltrate of mononuclear cells in gestational choriocarcinoma probably occurs in response to the presence of tumour antigens. The generally favourable effect of an infiltrate on the natural course of the disease and the response to chemotherapy suggest that it contributes to tumour cell death. It may be regarded as an immunological response directed at tumour rejection.

Our thanks are due to Mr K. James and Mrs D. Phillips for technical assistance and to Mr G. Harwood for the photomicrographs. The work was supported in part by the Charing Cross Hospital Research Sub-Committee.

### REFERENCES

Bagshawe, K. D. (1963) Trophoblastic Tumours: Chemotherapy and Developments. *Br. med. J.*, ii, 1303.

Bagshawe, K. D. (1967*a*) Immunological Aspects of Trophoblast. *J. Obstet. Gynaec. Brit. Cwlth*, **74**, 829.

Bagshawe, K. D. (1967*b*) Gonadotrophin Excretion, Pelvic Arteriography and Treatment in Post-molar Trophoblastic Disease. *Proc. R. Soc. Med.*, **60**, 240.

Bagshawe, K. D. (1968) Tumour Growth and Anti-mitotic Action: the Role of Spontaneous Cell Losses. *Br. J. Cancer*, **22**, 698.

Bagshawe, K. D. (1969) *Choriocarcinoma. The Clinical Biology of Trophoblast and its Tumours.* London: Edward Arnold.

Bagshawe, K. D., Rawlins, G., Pike, M. & Lawler, Sylvia, D. (1971) ABO Blood-groups in Trophoblastic neoplasia. *Lancet*, i, 553.

Bardawil, W. A. & Toy, B. L. (1959) The Natural History of Choriocarcinoma: Problems of Immunity and Spontaneous Regression. *Ann. N.Y. Acad. Sci.*, **80**, 197.

Beer, A. E., Billingham, R. E. & Yang, S. L. (1972) Further Evidence Concerning the Auto-antigenic status of the Trophoblast. *J. Exp. Med.*, **135**, 1177.

Berg, J. W. (1962) Active Host Resistance to Breast Cancer. *U.I.C.C.*, **18**, 854.

Billingham, R. E. (1964) Transplantation Immunity and the Maternofoetal Relationship. *New Engl. J. Med.*, **270**, 667, 720.

Billingham, R. E. (1967) Transplantation Immunity and Trophoblast. In U.I.C.C. Monograph Series Vol. 3. *Conference on the Chemotherapy of Choriocarcinoma.* Baguio. Ed. J. F. Holland and M. M. Hreshchyshin. Berlin: Springer-Verlag. p. 9.

Black, M. M., Opler, S. R. & Speer, F. D. (1954) Microscopic Structure of Gastric Carcinomas and their Regional Lymph Nodes in Relation to Survival. *Surg. Gynec. Obstet.*, **98**, 725.

Black, M. M., Opler, S. R. & Speer, F. D. (1956) Structural Representation of Tumour-host Relationships in Mammary Carcinoma. Biologic and Prognostic Significance. *Am. J. clin. Path.*, **26**, 250.

Bradbury, S., Billington, W. D., Kirby, D. R. S. & Williams, E. A. (1969) Surface Mucin of Human Trophoblast. *Am. J. Obstet. Gynec.*, **104**, 416.

Breyere, E. J. (1964) Maternal Immunologic Tolerance. *Med. Ann. D.C.*, **33**, 93.

Cochran, A. J. (1969) Histology and Prognosis in Malignant Melanoma. *J. Path.*, **97**, 459.

Collins, D. H. & Pugh, R. C. B. (1965) *The Pathology of Testicular Tumours.* London: Livingstone.

Currie, G. A. & Bagshawe, K. D. (1967) The Masking of Antigens on Trophoblast and Cancer Cells. *Lancet*, i, 708.

Currie, G. A., van Doorninck, W. & Bagshawe, K. D. (1968) Effect of Neuraminidase on the Immunogenicity of Early Mouse Trophoblast. *Nature, Lond.*, **219**, 191.

Dameshek, W. (1963) " Immunoblasts " and " Immunocytes "—an Attempt at a Functional Nomenclature. *Blood*, **21**, 243.

Dowling, E. A. (1957) Choriocarcinoma and Hydatidiform Mole as Tissue Transplants. *Sth. med. J., Nashville*, **50**, 211.

Dunnill, M. S. (1968) Quantitative Methods in Histology. In *Recent Advances in Clinical Pathology*, Series V. Ed. S. C. Dyke. London: Churchill. p. 401.

Elston, C. W. (1969) Cellular Reaction to Choriocarcinoma. *J. Path.*, **97**, 261.

Elston, C. W. (1970) M.D. Thesis, University of London.

Gowans, J. L. (1965) The Role of Lymphocytes in the Destruction of Homografts. *Br. med. Bull.*, **21**, 106.

Hackett. E. & Beech, M. (1961) Immunological Treatment of a Case of Choriocarcinoma. *Br. med. J.*, ii, 1123.

Hamlin, I. M. (1968) Possible Host Resistance in Carcinoma of the Breast: a Histological Study. *Br. J. Cancer*, **22**, 383.

Haskova, V. (1962) Transplantation Non-antigenicity of the Foetal Placenta. *Nature, Lond.*, **193**, 278.

HIRSCH, H. M. (1962) Some Aspects of the Problem of Immunity against Transplanted and Spontaneous Tumours. *Bact. Rev.*, **26**, 336.

ILIYA, F. A., WILLIAMSON, S. & AZAR, H. A. (1967) Choriocarcinoma in the Near East. *Cancer, N.Y.*, **20**, 144.

KIRBY, D. R. S., BILLINGTON, W. D., BRADBURY, S. & GOLDSTEIN, D. J. (1964) Antigen Barrier of the Mouse Placenta. *Nature, Lond.*, **204**, 548.

LALA, P. K. (1971) Studies on Tumour Cell Population Kinetics. In *Methods in Cancer Research*, Vol. 6. Ed. H. Busch. New York: Academic Press.

LAUDER, I. & AHERNE, W. (1972) The Significance of Lymphocytic Infiltration in Neuroblastoma. *Br. J. Cancer*, **26**, 321.

LAURENCE, D. J. R. & NEVILLE, A. MUNRO (1972) Foetal Antigens and their Role in the Diagnosis and Clinical Management of Human Neoplasms: a Review. *Br. J. Cancer*, **26**, 335.

LAWLER, S., KOUDA, P. T. & BAGSHAWE, K. D. (1971) The HL-A System in Trophoblastic Neoplasia. *Lancet*, ii, 834.

LEWIS, J. L. & TERASAKI, P. I. (1971) HL-A Leukocyte Antigen Studies in Women with Gestational Trophoblastic Neoplasms. *Am. J. Obstet. Gynec.*, **111**, 547.

MELLORS, R. C. (1966) Immunocytes and Immunoglobins. *Blood*, **27**, 871.

PARK, W. W. (1971) *Choriocarcinoma. A Study of its Pathology.* London: Wm. Heinemann.

PIERCE, G. B. JR & MIDGLEY, A. R. JR (1963) The Origin and Function of Human Syncytiotrophoblastic Giant Cells. *Am. J. Path.*, **43**, 153.

PREHN, R. T. (1960) Specific Homograft Tolerance Induced by Successive Matings and Implications Concerning Choriocarcinoma. *J. natn. Cancer Inst.*, **25**, 883.

SCOTT, J. S. (1962) Choriocarcinoma: Observations on the Etiology. *Am. J. Obstet. Gynec.*, **83**, 185.

SIMMONS, R. L. & RUSSELL, P. S. (1962) The Antigenicity of Mouse Trophoblast. *Ann. N.Y. Acad. Sci.*, **99**, 717.

SUTHERLAND, A. M. (1951) An Unusual Case of Chorionepithelioma of the Uterus. *J. Obstet. Gynaec. Br. Emp.*, **58**, 29.

WAKSMAN, B. H. (1960) A Comparative Histopathological Study of Delayed Hypersensitivity Reactions. In *Cellular Aspects of Immunity.* Ciba Fdn Symp. Ed. G. E. W. Wolstenholme and M. O'Connor. London. p. 280.